# Light-Mediated Enhancement of Carbon Sequestration in Engineered *Spathiphyllum*

**DOI:** 10.1021/acsenvironau.5c00268

**Published:** 2026-03-09

**Authors:** Yi-Jun Wang, Amit Kumar Sharma, Shu-Mei Wang, Fei Pan, Yen-Hsun Su

**Affiliations:** † Department of Materials Science and Engineering, 34912 National Cheng Kung University, No. 1, Daxue Road, East District, Tainan City 701, Taiwan; ‡ Department of Bio-Industry, 33561 National Taiwan University, No. 1, Sec. 4, Roosevelt Road, Taipei City 106, Taiwan; § Department of Health Science and Technology, 27219 ETH Zürich, Universitätstrasse 2, Zürich 8092, Switzerland

**Keywords:** carbon sequestration, glowing plants, phosphor, calcium sulfide, photosynthetic efficiency

## Abstract

Carbon sequestration is essential to mitigate climate change and atmospheric carbon dioxide levels. Utilizing indoor plants, such as *Spathiphyllum* (Peace Lily), is desirable for sustainable living, thanks to their carbon sequestration characteristics and ability to remove air toxins and adjust humidity. In this study, a luminescent energy storage material, calcium sulfide doped with europium and dysprosium (CaS:Eu,Dy) phosphor, was synthesized and applied to the surface of *Spathiphyllum* (Peace Lily) leaves to improve its photosynthetic efficiency and carbon sink potential. This phosphor absorbs incident visible light and converts short-wavelength light to long-wavelength light. When excited by 514 nm light, the CaS:Eu,Dy phosphor emits a red light at 652 nm. Additionally, CaS:0.5%Eu,0.25%Dy has the best luminescence duration compared to other dopants, with a relaxation time of 2.392 s. To prevent the phosphor from hygroscopic degradation in the air, a SiO_2_ coating was applied to the material’s surface, increasing the phosphor’s lifespan and preventing environmental damage. Based on the chlorophyll fluorescence induction OJIP curve, it was observed that using the phosphor on the leaves of Peace Lily does not affect the plant’s physiological condition, and the plant remains healthy. Furthermore, compared to untreated leaves, the photosynthetic efficiency of treated Peace Lily leaves could increase by 42%, resulting in an additional carbon sequestration of about 0.045 mol of CO_2_ per square meter of leaves per day. This also allows the Peace Lily to emit red light in the dark, thereby enhancing its ornamental value as an indoor plant.

## Graphical abstract

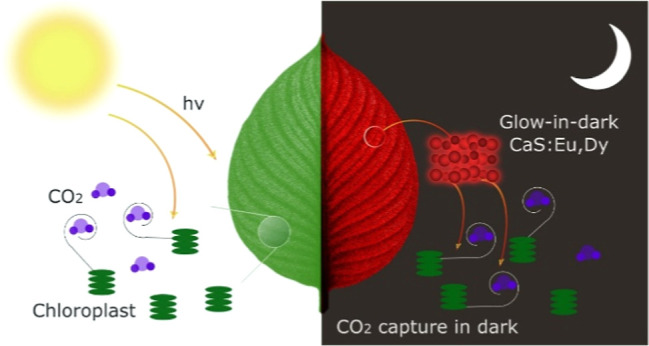

## 1 Introduction

Continuous advancements in science and technology have made human life significantly convenient and efficient. However, industrialization, urbanization, and large scale energy consumption have led to a steady increase in CO_2_ emissions, posing a serious global challenge to the environment and ecosystem.^1^ As one of the primary greenhouse gases, excessive CO_2_ emissions are considered a major driver of climate change and global warming, leading to more frequent extreme weather events, accelerated glacier melting, rising sea levels, and biodiversity loss.^2,3^ These consequences threaten the sustainable development of the human society. In response to this global crisis, the international community has been actively seeking strategies to mitigate climate change.^4^

Against this backdrop, the 2050 net-zero emission target has emerged as a cornerstone of global climate governance.^5^ Many nations, including the European Union, the United Kingdom, and Japan, have pledged to reach net-zero emissions by 2050.^6^ Realizing these commitments relies on a range of efforts, including advanced clean energy technologies,^7^ improving energy efficiency,^8^ and developing carbon capture and storage (CCS) technologies.^9^ As a critical strategy for achieving net-zero emissions, carbon neutrality focuses on offsetting human-induced CO_2_ emissions through measures such as afforestation and carbon capture technologies.^10,11^ Although current carbon capture technologies are theoretically feasible, their high costs, low conversion efficiency, and potential environmental impacts limit large-scale implementation.^12^ Therefore, developing efficient, sustainable, and ecofriendly carbon capture technologies has become a key focus for both academia and industry.

Our research addresses this need by drawing inspiration from natural plant photosynthesis and exploring an innovative carbon capture method. Photosynthesis is a process in which plants fix CO_2_ into carbohydrates and oxygen with high efficiency and zero pollution, making it an ideal model for carbon sequestration.^13^ However, the efficiency of photosynthesis is constrained by the duration of sunlight exposure and its intensity. In suboptimal light conditions or regions with limited sunlight, the carbon-fixing capabilities of plants are not fully utilized.^14^ To overcome this limitation, we propose using phosphors as energy storage materials to extend the light cycle and enhance the light utilization efficiency of plants. Phosphors are materials capable of emitting light for a certain period after being excited by a light source, effectively extending the illumination required for photosynthesis.^15,16^ The application of phosphors in plants has been shown in the literature to promote plant growth and enhance photosynthesis rates.^17–19^ For instance, Wang et al. used CaS:Eu^2+^, CaBr_2_, and CaF_2_ phosphors as agricultural greenhouse films, demonstrating excellent weather resistance, higher solar conversion efficiency, and longer photoconversion lifetimes. Increased red light intensity and prolonged exposure significantly improved the yields of crops such as strawberries, tomatoes, and peppers.^20^ Additionally, Wang et al. utilized GYIG:Cr^3+^ to fabricate LEDs for lighting *Epipremnum aureum*, conducting six different tests that verified the phosphor’s growth-promoting effects in plant.^21^ Another study by Wu et al. blended Sr_2_Si_5_N_8_:2%Eu^2+^ phosphors with polyethylene to create photoconversion films. These films converted blue-violet light into red light and, when applied to vegetable cultivation, significantly increased the biomass of Chinese cabbage compared to that of the control group. Furthermore, carotenoid content increased by 45.6%, and total chlorophyll content rose by 29.6%, effectively promoting plant growth and enhancing photosynthesis efficiency.^22^

Chlorophyll absorbs light primarily in the blue-violet (Soret band 400–500 nm) and red-orange (Q-band 600–700 nm) regions of the visible-light spectrum.^23^ Therefore, we focus on developing phosphors that emit light within these wavelengths, ensuring that plants absorb the light efficiently. Alkaline-earth sulfides (AESs), known for their chemical stability and excellent luminescent properties, are promising candidates for phosphor materials.^24^ Among them, calcium sulfide (CaS) is a wide-bandgap material that can be doped with various rare-earth elements to adjust emission wavelengths, making it our preferred choice.^25^ For instance, europium (Eu)-doped CaS emits high-intensity red light at around 650 nm,^26^ aligning well with the red absorption band of chlorophyll. Dysprosium (Dy) codoping can further extend the afterglow duration, allowing the phosphor to continue emitting light after the excitation ceases.^27^ *Spathiphyllum* (commonly known as Peace Lily) was chosen as it is a widely used ornamental plant for indoor greening.^28^ It is known for its lush green leaves and elegant white inflorescences. As per NASA, Peace Lily is ranked among the top ten air-purifying plants capable of removing harmful indoor gases such as formaldehyde and benzene.^29^ Its strong adaptability and high photosynthetic efficiency make it an ideal subject for studying phosphor-enhanced photosynthesis.^30,31^

This research aims to develop phosphor-based energy storage material that can be applied to plant leaves to extend the light cycle and improve photosynthetic efficiency. We have designed a comprehensive set of experiments, including synthesis and optical characterization of the phosphor and its application on Peace Lily leaves, systematically evaluating the impact of the phosphor on the plant’s carbon fixation capabilities. In the future, we aim to integrate this technology into the broader framework of the 2050 net-zero emissions goal, promoting its applicability in indoor plant-based carbon neutrality initiatives and extending its use to agriculture and ecological restoration.

## 2 Experimental Section

### 2.1 Materials

All chemical reagents were of analytical grade and used without further purification. Calcium chloride dihydrate (≥99%, CaCl_2_·2H_2_O) was purchased from Fisher Scientific. Europium­(III) nitrate hexahydrate (99.9%, Eu­(NO_3_)_3_·6H_2_O) and Dysprosium­(III) nitrate pentahydrate (99.9%, Dy­(NO_3_)_3_·5H_2_O) were obtained from Thermo Scientific and Alfa Aesar, respectively. Sodium sulfide nonahydrate (≥98%, Na_2_S·9H_2_O) was supplied by Macklin. 3-Mercapto-1,2-propanediol (90%) was procured from Thermo Scientific, while ethanol (≥99.9%) and ammonium hydroxide (NH_4_OH) were sourced from Echo Chemical. Tetraethyl orthosilicate (TEOS) was acquired from Seedchem. To maintain a consistent reaction environment, ethanol (≥99.9%) was used as the solvent.

### 2.2 Synthesis

In this study, CaS:Eu^2+^,Dy^3+^ phosphors were synthesized using a wet chemical coprecipitation method. Initially, precursor solutions were prepared based on stoichiometric ratios by dissolving calcium chloride (CaCl_2_·2H_2_O), europium nitrate (Eu­(NO_3_)_3_·6H_2_O), and dysprosium nitrate (Dy­(NO_3_)_3_·5H_2_O) in ethanol. These solutions were then mixed and stirred thoroughly to achieve homogeneity. Subsequently, a sodium sulfide (Na_2_S·9H_2_O) ethanol solution was slowly added dropwise to the mixture with constant stirring at room temperature for 16 h to ensure a complete reaction. After centrifugation and drying, CaS was doped with Eu^3+^ and Dy^3+^ (1, CaS:0.5%Eu^3+^,0.1%Dy^3+^; 2, CaS:0.5%Eu^3+^,0.25%Dy^3+^; and 3, CaS:0.5%Eu^3+^,0.5%Dy^3+^) and obtained in powder form.

To enhance the performance of the phosphor and reduce Eu^3+^ to the more luminescent Eu^2+^ state, the CaS powder was subjected to calcination in a tubular furnace. The furnace atmosphere comprised a mixture of argon (95%) and hydrogen (5%), and the material was heated to 900 °C for 4 h. This reduction process successfully reduced Eu^3+^ to Eu^2+^, resulting in a CaS:Eu^2+^,Dy^3+^ phosphor with excellent luminescent properties.

Given that CaS-based materials are prone to hygroscopic degradation when exposed to air, the phosphor surface was coated with silicon dioxide (SiO_2_) to improve its moisture resistance and environmental stability. Specifically, the CaS:Eu^2+^,Dy^3+^ powder was dispersed in an ethanol solution, followed by adding 0.2 mL ammonia–water and 1.8 mL deionized water as catalysts and reaction media. 0.625 mL of tetraethyl orthosilicate (TEOS) was then added dropwise to the solution while stirring at room temperature for 12 h to ensure uniform deposition of the SiO_2_ layer on the phosphor surface. After the reaction, the resulting SiO_2_-coated CaS:Eu^2+^,Dy^3+^ (SiO_2_@CaS:Eu^2+^,Dy^3+^) was collected via centrifugation and washed thoroughly with water.

### 2.3 Applied to Peace Lily Leaves

A paint formulation was prepared by combining 0.1 g of SiO_2_@CaS:Eu^2+^,Dy^3+^ with 1 g of a biocompatible water-based resin. The preparation of this mixture was carefully designed to ensure uniform dispersion and high stability, allowing it to be effectively applied as a thin and consistent layer on the surface of Peace Lily leaves. This experimental setup is aimed at exploring the material’s potential applications in enhancing photosynthetic efficiency. As a highly efficient indoor plant, Peace Lily provides an innovative platform for integrating luminescent materials with biological systems, potentially improving light utilization for photosynthesis and enhancing the carbon dioxide fixation capacity.

### 2.4 Instrumentation

The structural characterization of the synthesized materials was performed using powder X-ray diffraction (XRD) on a Bruker-D8 DISCOVER diffractometer. Morphological and microstructural analyses were conducted using scanning electron microscopy (SEM, HITACHI SU8010) and transmission electron microscopy (TEM, JEOL JEM-2100F). Photoluminescence properties were measured by using a fluorescence spectrophotometer (Hitachi F-7000). Elemental composition and trace element quantification were analyzed by using an inductively coupled plasma mass spectrometer (ICP–MS, THERMO-ELEMENT XR). Surface charge measurements were conducted by using a zeta potential analyzer (OTSUKA ELSZ-2000). For assessment of the physiological status of leaves, chlorophyll fluorescence was measured with a Chlorophyll Fluorometer (Hansatech Handy PEA+), and the photosynthetic assimilation rate was evaluated by using a Portable Photosynthesis System (ADC BioScientific LCpro T).

## 3 Results and Discussions

The XRD spectra (Figure 1) reveal that the main peak positions of all three phosphors are essentially consistent and align with the standard CaS spectrum (JCPDF #08-0464). This indicates that doping with different concentrations of Dy has not significantly affected the crystal structure of CaS, and the introduction of Eu and Dy ions has not led to the formation of new phases, preserving the main crystal phase of CaS. Additionally, the diffraction peaks exhibit sharp and clear features, further confirming that the samples have a good crystal structure and high crystallinity.

**Figure 1:**
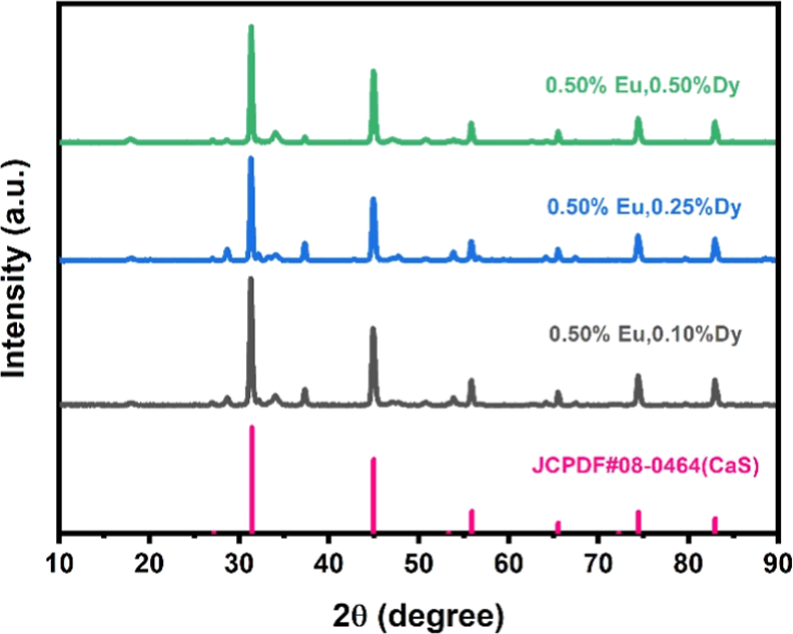
X-ray diffraction patterns of CaS:Eu,Dy phosphors compared with the standard CaS spectra.

The elemental proportions of Eu and Dy doped into the CaS structure were confirmed using the ICP–MS technique (Table 1). In the 0.50%Eu, 0.50%Dy-doped sample, the measured Eu concentration was 0.913% and Dy concentration was 0.707%; in the 0.50%Eu, 0.25%Dy-doped sample, the Eu concentration was 0.513% and Dy concentration was 0.212%; in the 0.50%Eu, 0.10%Dy-doped sample, the Eu concentration was 0.503% and Dy concentration was 0.072%. These results confirm that Eu and Dy have successfully doped into the CaS host lattice with the actual doping concentrations being consistent with the added amounts of chemical agents.

**Table 1:** Determination of Mass Percentage Concentration of Eu and Dy in Various Eu- and Dy-Doped Phosphors

sample	element	mass percentage concentration (%)
0.50% Eu, 0.50% Dy	Eu	0.913
	Dy	0.707
0.50% Eu, 0.25% Dy	Eu	0.513
	Dy	0.212
0.50% Eu, 0.10% Dy	Eu	0.503
	Dy	0.072

Figure 2 shows the morphology of the three phosphors characterized by SEM and TEM. It was observed that CaS:Eu,Dy exhibits a polygonal particle morphology with uniform particle size, clear and smooth edges, and tightly packed particles with a particle size of approximately 800 nm (Figure 2c). Furthermore, for CaS:Eu,Dy phosphors coated with SiO_2_, Figure 2b,d shows that spherical silica particles (approximately 150 nm) were attached to the surface of the phosphor particle, clearly indicating that the phosphors were successfully protected by silica.

**Figure 2:**
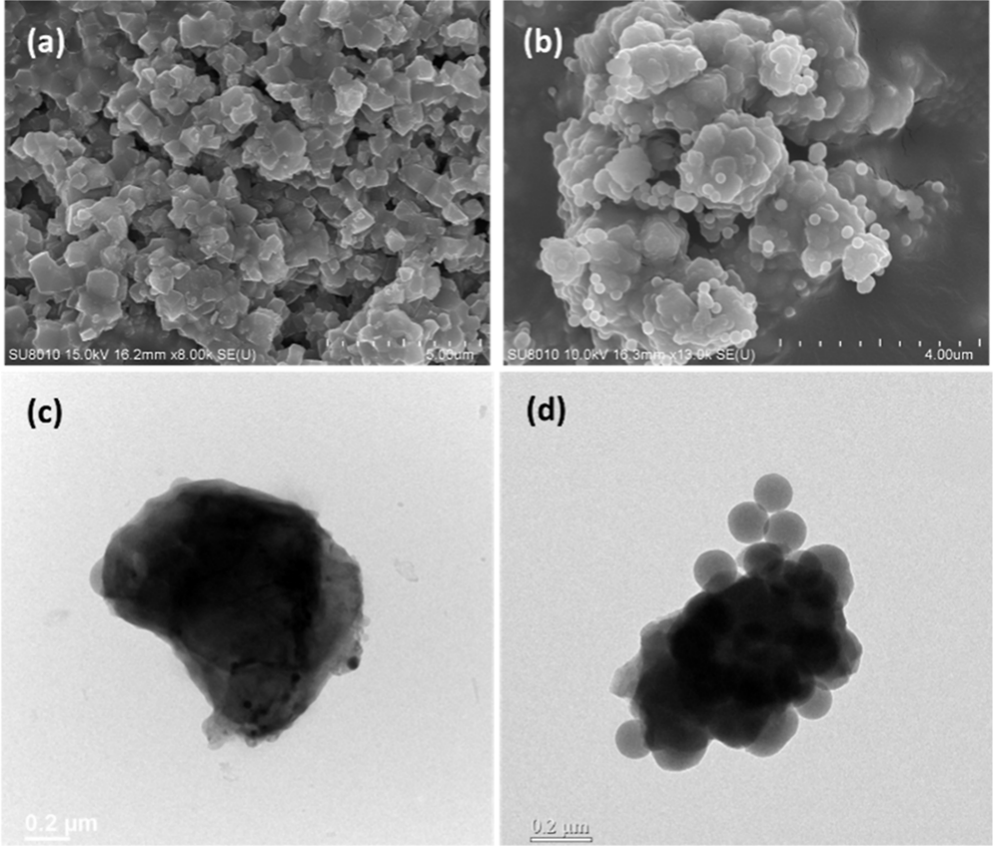
SEM images of (a) CaS:Eu,Dy and (b) SiO_2_@CaS:Eu,Dy. TEM images of (c) CaS:Eu,Dy and (d) SiO_2_@CaS:Eu,Dy.

The surface charge of the CaS:Eu,Dy phosphor material exhibits a negative zeta potential, likely due to sulfur ions on its surface, as shown in Figure 3. In contrast, the SiO_2_-coated phosphor shows a positive zeta potential, attributed to the positively charged SiO_2_ protection on the surface. Additionally, the absolute value of the zeta potential for the SiO_2_-coated material is relatively higher, indicating better dispersibility in ethanol.

**Figure 3:**
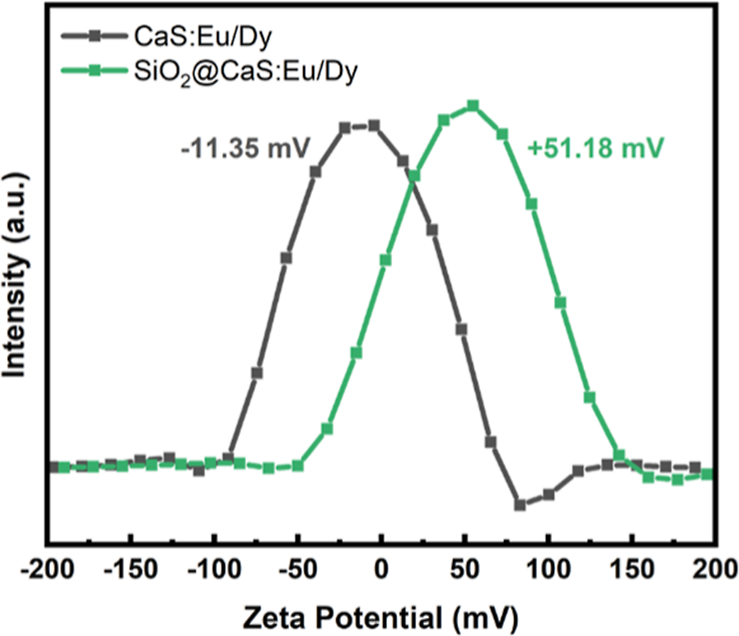
Zeta potential of CaS:Eu,Dy and SiO_2_@CaS:Eu,Dy.

The three phosphors’ photoluminescence excitation and emission spectra (Figure 4a) reveal broader excitation spectra between 450 and 600 nm, with a maximum excitation peak at 514 nm. The corresponding emission spectrum (Figure 4b) reveals narrow peaks between 600 and 700 nm, with a maximum emission peak at 652 nm corresponding to red light. The red emission peak is due to the transition of Eu^2+^ electrons from the 4f^6^5d^1^ excited state to the 4f^7^ ground state.^32^ Additionally, a comparative analysis of emission intensities revealed that the CaS sample doped with 0.5% Eu and 0.25% Dy demonstrated the highest emission intensity. Further, phosphorescence lifetime analysis showed that the relaxation times for different doping ratios followed the order (Figure 4b): 0.5% Eu, 0.25% Dy (2.392 s) > 0.5% Eu, 0.5% Dy (2.362 s) > 0.5% Eu, 0.1% Dy (2.152 s). This indicates that the CaS sample doped with 0.5% Eu and 0.25% Dy not only has the longest afterglow duration but also exhibits superior luminescence intensity. Overall, the doping ratio of 0.5% Eu to 0.25% Dy is identified as the optimal combination, making this material highly suitable as an efficient red-light-emitting phosphor.

**Figure 4:**
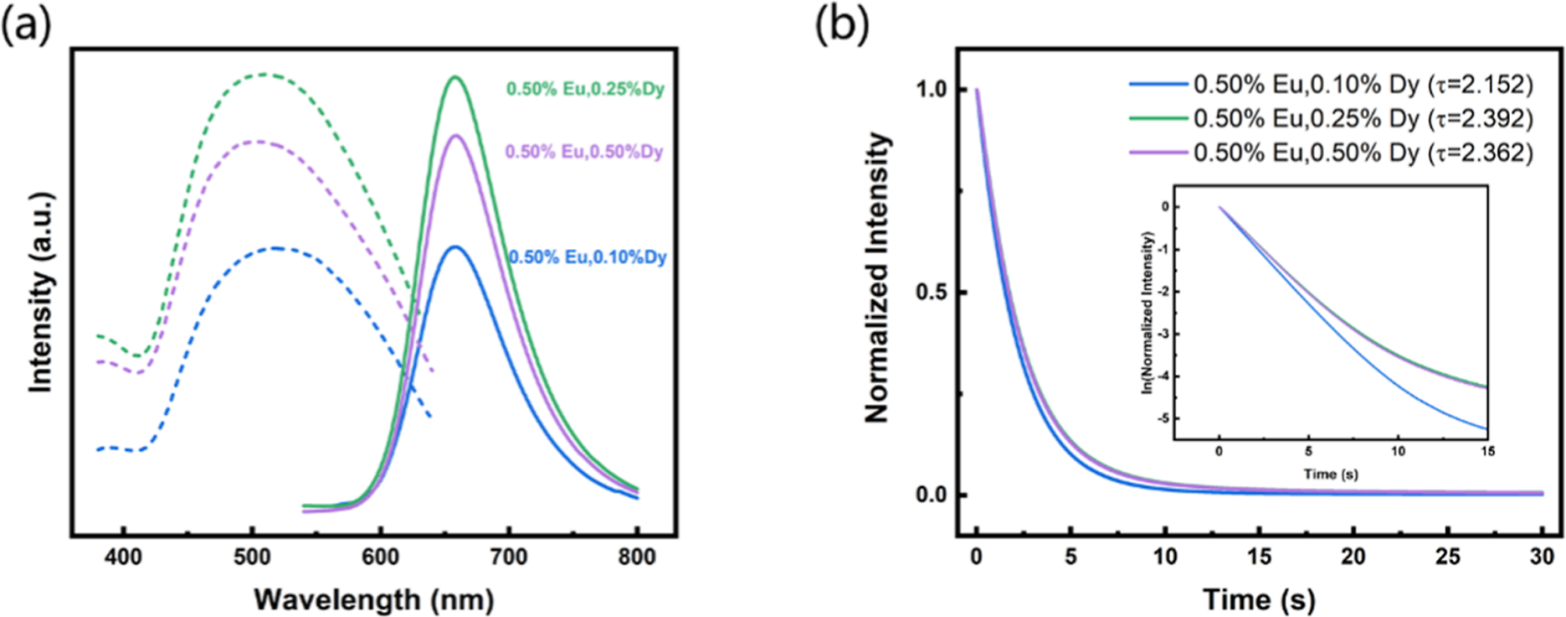
(a) Photoluminescence excitation (PLE) (dotted lines) and photoluminescence (PL) (solid lines) spectra and (b) phosphorescence lifetime spectra of CaS:0.5%Eu,0.1%Dy, CaS:0.5%Eu,0.25%Dy, and CaS:0.5%Eu,0.5%Dy.

Based on the photoluminescence spectra, CaS:0.5%Eu,0.25%Dy phosphor material was chosen to paint the surface of Peace Lily leaves, and the corresponding excitation-induced emission was observed using a Fluorescence microscope. Figures 5 and S1 (Supporting Information) evidently reveal the uniform distribution of the phosphor material on the leaf surface, emitting a prominent red fluorescence. It was observed that the CaS:Eu,Dy phosphor material stably adheres to the surface of the plant leaves, owing to the SiO_2_ protection layer.^33,34^ Furthermore, the intensity of the red fluorescence reflects the material’s high energy conversion efficiency under excitation by a light source, highlighting its potential for light energy conversion applications. More importantly, this efficient fluorescence characteristic provides an additional and effective light source for chloroplasts, potentially enhancing photosynthetic efficiency and thereby improving the plant’s carbon fixation capacity.

**Figure 5:**
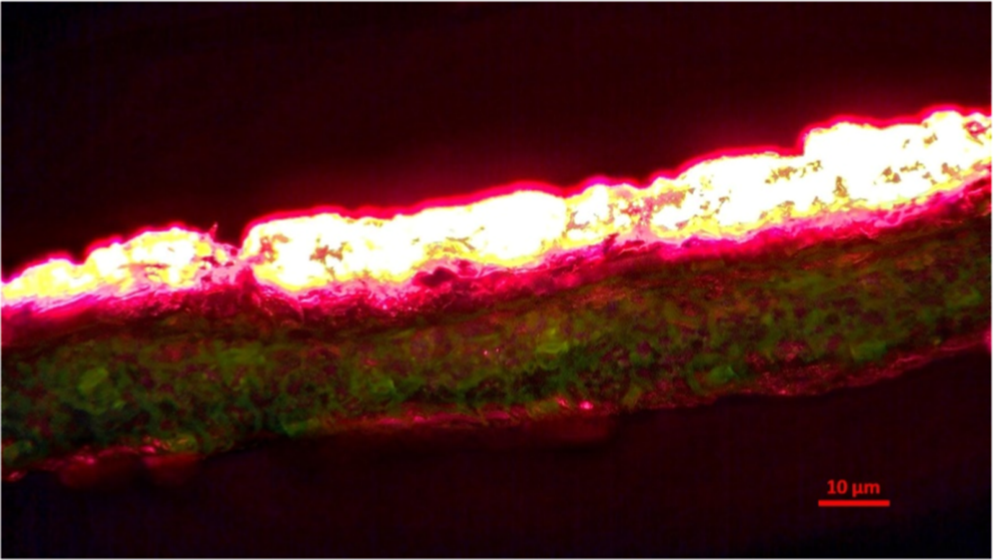
Fluorescence microscopy image of Peace Lily leaf surface painted with CaS:0.5%Eu,0.25%Dy phosphor.

Notably, upon excitation, the afterglow duration can be observed for more than 5 min, rendering the photon fluxes over 7 to 10 min, while a dark phase may be below the plant’s light compensation point. Thus, spectral downconversion, incident visible light to emitted red light, is considered the key mechanism of increased photosynthetic efficiency. It is seen that the phosphor coating effectively absorbs UV/ambient light and converts it into red light with a central wavelength of 652 nm. The emitted light aligns with the absorption peaks of chlorophyll, enhancing in situ light quality during a 12 h photoperiod—resulting in a 42% increased photosynthetic rate. The afterglow emission of the phosphor redistributes a fraction of incident photons over short time scales, potentially smoothing rapid irradiance fluctuations at the leaf surface.^35^ This temporally extended but low-intensity emission may contribute to more stable excitation conditions for the photosystems during brief light gaps.

The chlorophyll fluorescence performance of Peace Lily leaves painted with CaS phosphors doped with various concentrations of Eu and Dy was measured by using a chlorophyll fluorometer (Figure 6a). According to the JIP-test parameters,^36^ the leaves painted with the phosphor material showed significant improvement compared to the control group―the best performance was observed in leaves painted using CaS doped with 0.5% Eu and 0.25% Dy. Specifically, the experimental group exhibited a lower dissipation energy (DI_0_/RC) than the control group, indicating a reduced amount of absorbed light released as heat and an increase in the proportion of light energy utilized for photochemical reactions. Additionally, it was observed that the painted leaves after the seventh day exhibited the highest total performance index (PI_total_), indicating the most optimal overall photosynthetic efficiency. This finding suggests that by seventh day, the leaves had adapted to the presence of the phosphor on their surface (Figure S2).

**Figure 6:**
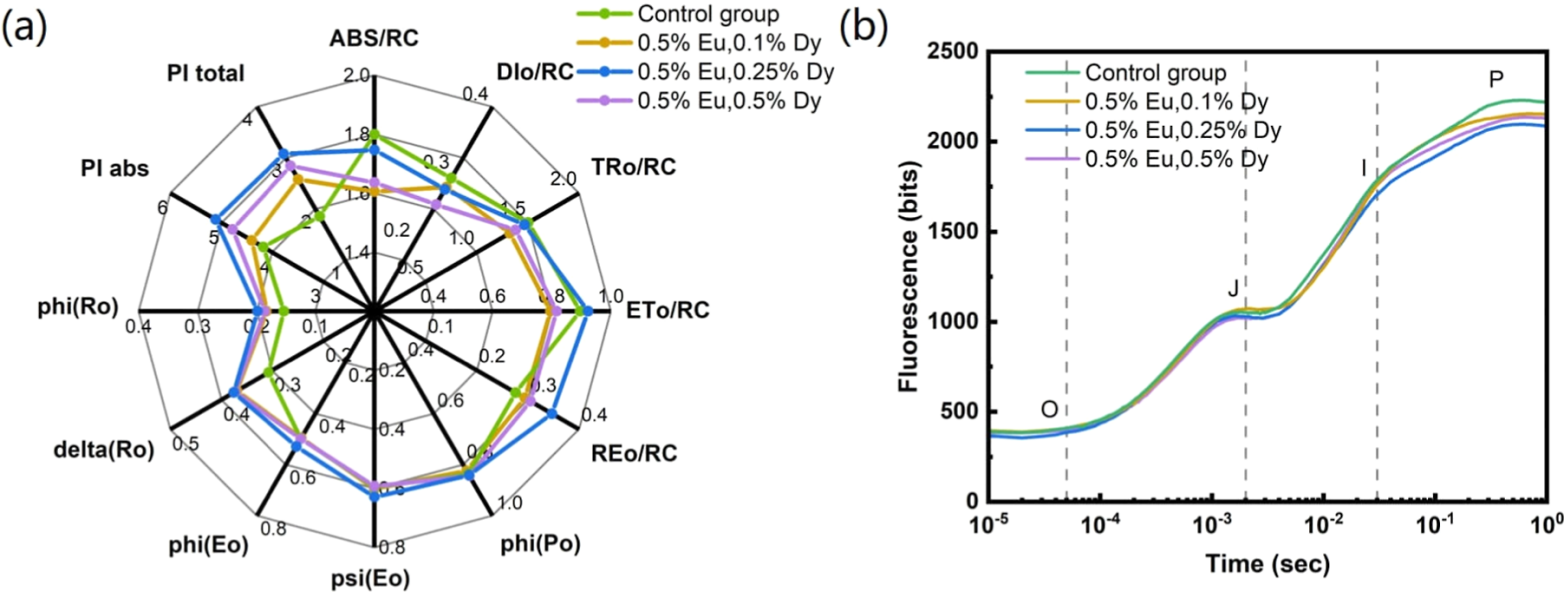
(a) JIP-test parameters and (b) OJIP curve of Peace Lily leaves painted with CaS:0.5%Eu,0.1%Dy, CaS:0.5%Eu,0.25%Dy, and CaS:0.5%Eu,0.5%Dy.

Furthermore, the electron transport to PS-I (RE_0_/RC) was significantly higher in the group with 0.5% Eu and 0.25% Dy doping, along with a noticeable increase in performance indices PI_abs_ and PI_total_. Analysis of the OJIP fluorescence transient curve (Figure 6b) revealed that CaS doped with 0.5% Eu and 0.25% Dy exhibited the lowest maximum fluorescence value (Fm). JIP-test parameters suggest a decrease in energy released as chlorophyll fluorescence and a corresponding increase in energy directed toward photochemical reactions, leading to an enhanced photochemical efficiency.

The transient chlorophyll fluorescence analysis confirms that painting Peace Lily leaves with CaS:Eu,Dy materials does not cause observable negative effects, allowing the plants to remain healthy while exhibiting improved photochemical efficiency. Among all tested materials, CaS doped with 0.5% Eu and 0.25% Dy stands out, showing superior performance and effectively enhancing the leaves’ ability to utilize light energy for photosynthesis.

The effects of Eu- and Dy-doped CaS phosphors on the photosynthetic rate were assessed, as shown in Figure 7. The results revealed that the phosphor-painted leaves exhibited a significantly higher photosynthetic rate compared to the control group. In particular, CaS doped with 0.5% Eu and 0.25% Dy exhibited optimal photosynthetic performance of the leaves. As per the experimental data recorded daily for seven days (Table 2), each square meter of the leaf surface could absorb 0.152 mol of CO_2_, an additional 0.045 mol of CO_2_ compared to the control group, representing a 42% increase. These results confirm that painting the Peace Lily leaves with Eu- and Dy-doped CaS phosphor significantly enhances the photosynthetic rate and effectively strengthens its carbon sequestration ability. In particular, when CaS is doped with 0.5% Eu and 0.25% Dy, the carbon sequestration effect is optimal.

**Figure 7:**
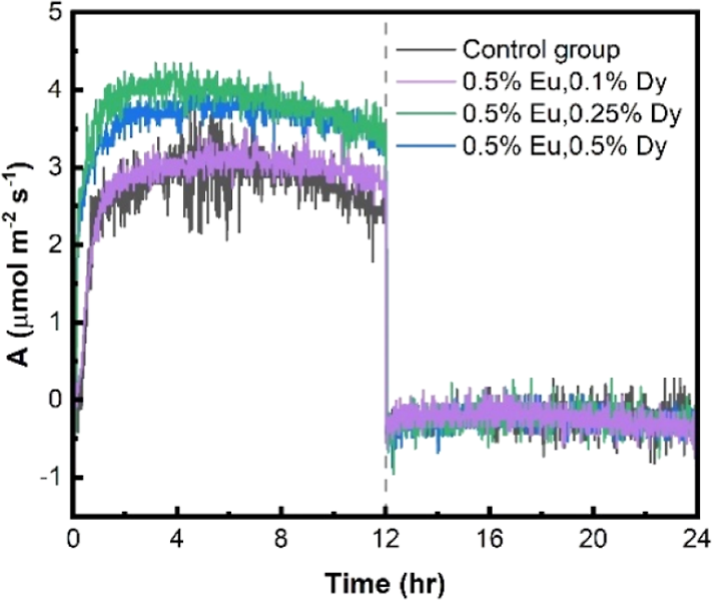
Photosynthetic rate of Peace Lily leaves with different material concentrations (CO_2_ = 400 ppm, P.A.R. = 250 μmol m^–2^ s^–1^) (0–12 h: in light, 12–24 h: in dark).

**Table 2:** Photosynthetic Rate (*A*) Data of CaS Doped with Different Eu and Dy Concentrations

	average *A* (μmol m^–2^ s^–1^)	*A* (mol m^–2^ day^–1^)	Δ*A*
control group	1.23	0.107	-
0.5%Eu,0.1%Dy	1.30	0.113	+6%
0.5%Eu,0.25%Dy	1.76	0.152	+42%
0.5%Eu,0.5%Dy	1.65	0.143	+35%

The stable adherence of the phosphor composite on the plant leaves and subsequent effects on its physiology and growth were evaluated through a 28 day continuous monitoring period. Figures S4 and S5a show the overall PSII efficiency, which overlaps with the control group. A lower relative variable fluorescence at the J-step (Vj) and I-step compared to the control group (Figure S5b,c) indicates no blockages to electron transport and thus less stress to the plant. A larger Sm (electron acceptor pool at PSII) and N (PSII stability) indicate greater capacity for electron transport compared to the control group (Figure S5d,e).

Figures S6a,b show absorption and dissipation per reaction center (ABS/RC and Dio/RC), respectively. A lower value compared with the control group indicates appropriate energy absorption and lower energy loss. Figure S6c reflects PSII efficiency in capturing excitons, while Figures S6d,e indicate the electron transport from PSII to PSI. The overlapping values with the control group validate healthy plant growth.

Figure S7a shows that phi­(Po) overlaps with the control group, indicating stable PSII activity. psi­(Eo) and phi­(Eo) verify that light energy is converted into useable electron flow (Figure S7b,c), while delta­(Ro) and phi­(Ro) indicate stable PSI activity (Figure S7d,e). Figure 8a,b shows PI_abs_ and PI_total_ indicators, which are higher than those of the control group. This reflects on the efficiency of the entire photosynthetic chain performance, including PSI. The carbon fixation in phosphor-treated groups was calculated to be 8.512 mol of CO_2_ m^–2^ higher than the control group, tabulated in Table S1.

**Figure 8:**
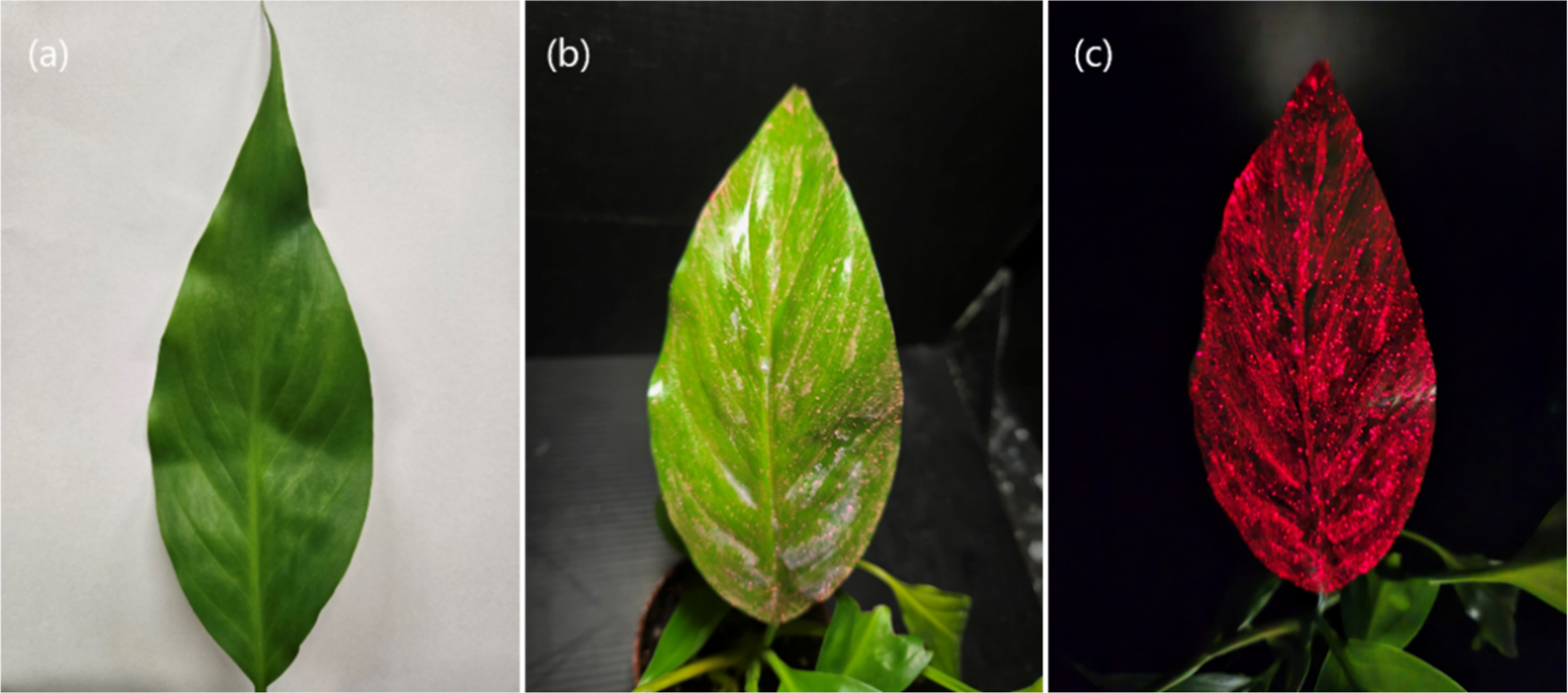
Ambient light images of Peace Lily leaf. (a) Untreated leaf, (b) leaf painted with CaS:0.5%Eu,0.25%Dy material, exposed to visible light, and (c) leaf painted with CaS:0.5%Eu,0.25%Dy material emitting red light in darkness.

In addition to enhancing the carbon sequestration efficiency of Peace Lily leaves painted with the CaS:Eu,Dy material, this material also exhibits energy storage and delayed luminescence properties. After exposure to sunlight, visible light, or a light source of specific excitation wavelength for a certain period, the leaf surface emits red light in a dark environment, even after the light source is turned off (Figure 8). This luminescence phenomenon not only demonstrates the material’s functionality but also introduces a novel aesthetic and therapeutic experience for plant applications. Plants with this nighttime self-luminescent characteristic significantly enhance the ornamental value of indoor greenery, opening up new possibilities for innovation and promotion in the horticulture industry. Furthermore, this integration of environmental sustainability and artistic design highlights the potential for a harmonious fusion of technology and nature.

## 4 Conclusion

In this study, the CaS:Eu,Dy phosphor was successfully synthesized, emitting red light at 652 nm. Additionally, CaS:0.5%Eu,0.25%Dy exhibited optimal phosphorescence. OJIP test results show that the phosphor material applied on Peace Lily leaves did not affect the plant’s physiological condition, and the plant remained healthy during the monitoring period of 28 days. Furthermore, treated leaves could increase carbon sequestration by approximately 0.152 mol CO_2_ per square meter per day compared to untreated control leaves. The relative change in carbon fixation was calculated to be 8.512 mol CO_2_ m^–2^. This effectively enhances the plant’s carbon sequestration ability and its potential use as carbon sink. The red emission from the plant leaves further enhances its ornamental value and aesthetic appeal in indoor spaces.

## Supplementary Material



## Notes

Fluorescence microscopy image of Peace Lily, OJIP curves, photosynthetic rate of Peace Lily after treatment with phosphor, tabulated photosynthetic rate, quantum yield of chlorophyll, and plant health performance indicators studied for 28 days (PDF)
