# Supplementary material for: Light-Mediated Enhancement of Carbon Sequestration in Engineered Spathiphyllum
Source: ACS Environ Au. 2026 Mar 9;6(3):449–55. doi: 10.1021/acsenvironau.5c00268 (PMC13195465; doi:10.1021/acsenvironau.5c00268)
Supplement: Supplementary file 1 [file vg5c00268_si_001.pdf]

Supporting Information

Light-Mediated Enhancement of Carbon Sequestration in Engineered Spathiphyllum

Yi-Jun Wang[a], Amit Kumar Sharma[a], Shu-Mei Wang[b], Fei Pan[c]*, Yen-Hsun Su[a]*

[a] Department of Materials Science and Engineering, National Cheng Kung University, No. 1, Daxue Road, East District, Tainan City 701, Taiwan

[b] Department of Bio-Industry, National Taiwan University, No. 1, Sec. 4, Roosevelt Road, Taipei City 106, Taiwan

[c] Department of Health Science and Technology, ETH Zürich, Universitätstrasse 2, 8092 Zürich, Switzerland

*Correspondence: fepan@ethz.ch, phoenix.pan@tum.de (F. P); yhsu@mail.ncku.edu.tw (Y.H. S)

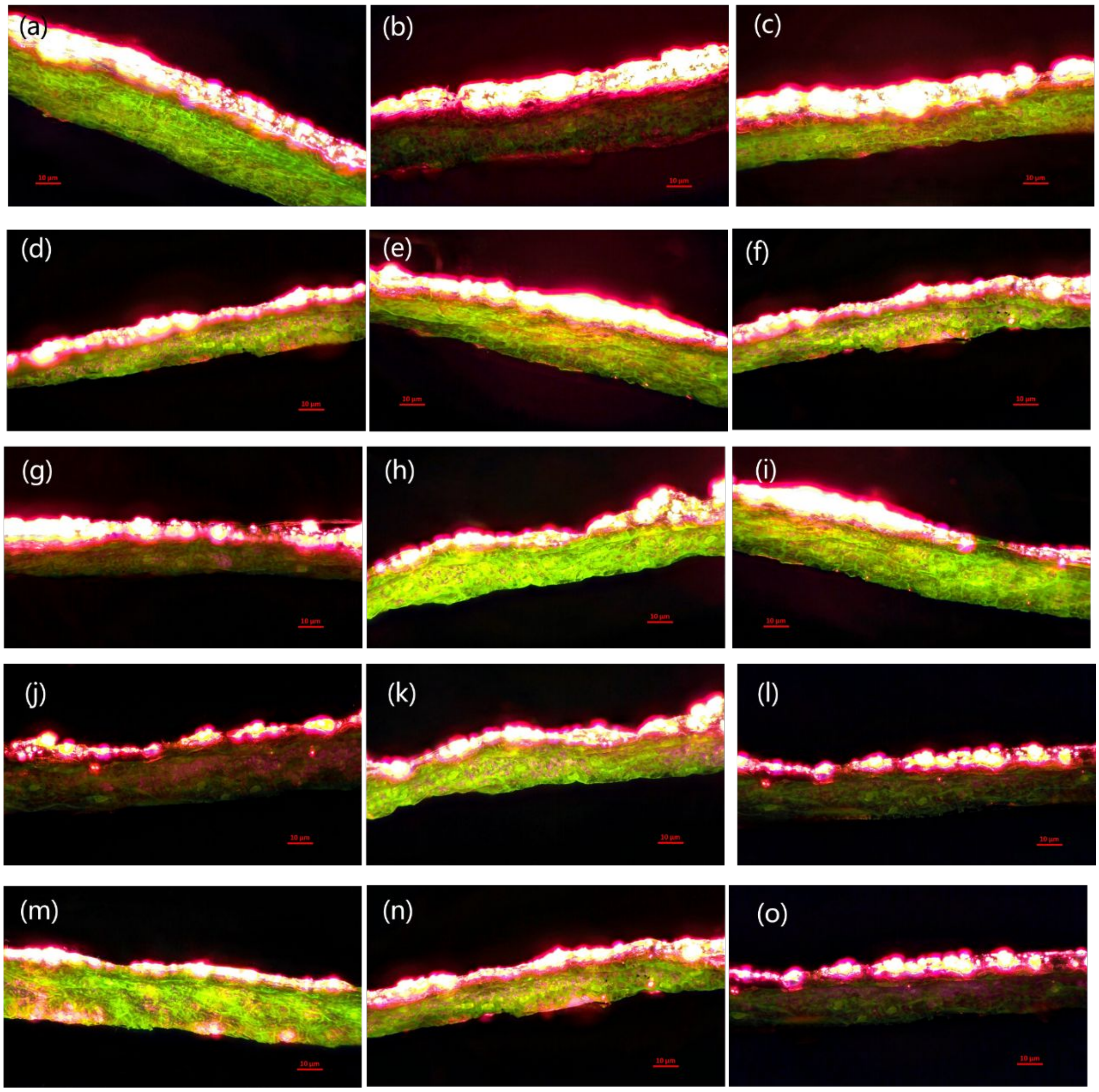

Figure S1. Fluorescence microscopy images of Peace Lily leaf surfaces over time (Day 1, 3, 5, 7, and 9) treated with different CaS:Eu,Dy compositions.

(a, d, g, j, m) CaS:0.5%Eu,0.1%Dy;

(b, e, h, k, n) CaS:0.5%Eu,0.25%Dy;

(c, f, i, l, o) CaS:0.5%Eu,0.5%Dy.

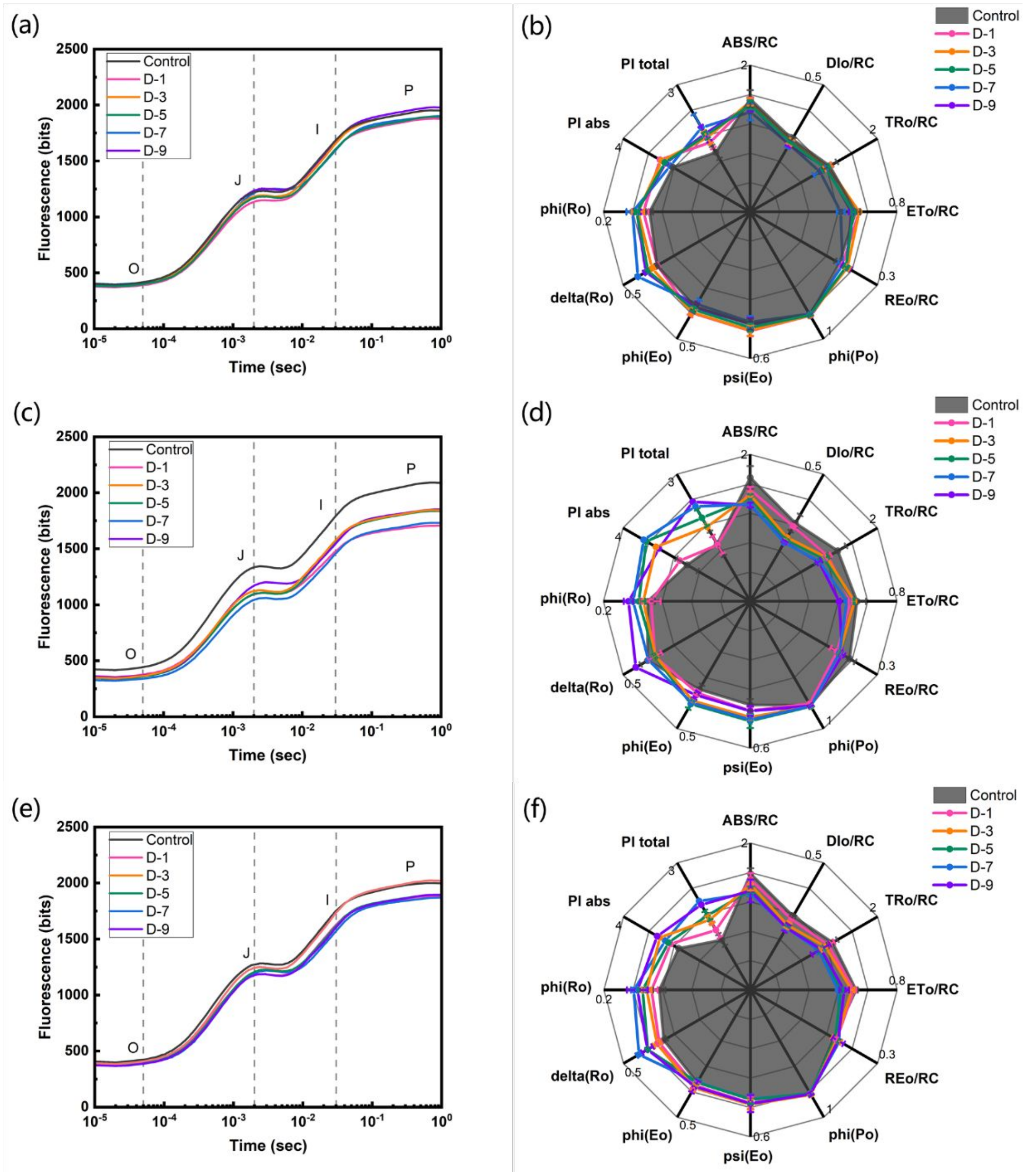

Figure S2. OJIP curves and JIP-test parameters of Peace Lily leaves treated with CaS:Eu,Dy compositions over time.

(a, b) CaS:0.5%Eu,0.1%Dy;

(c, d) CaS:0.5%Eu,0.25%Dy;

(e, f) CaS:0.5%Eu,0.5%Dy.

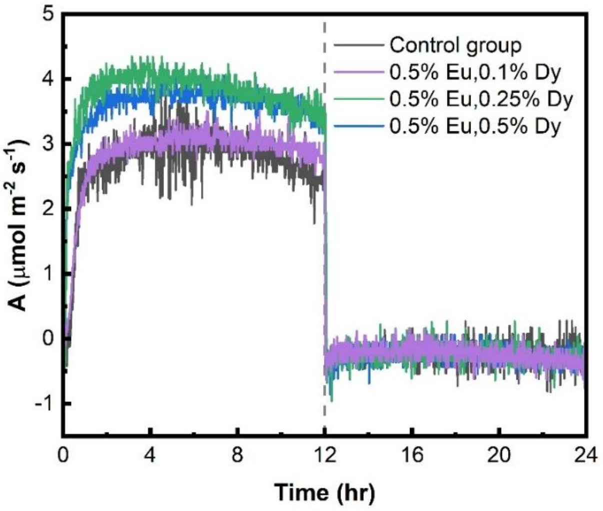

Figure S3: Photosynthetic rate calculated after 7 days under light (0–12 hours) and dark (12–24 hours) conditions with CaS:0.5% Eu and Dy (0.1%, 0.25%, 0.5%) is compared with that of the control group.

Table S1: Tabulated data on photosynthetic rate and carbon fixation in control and phosphor treated groups.

	Net photosynthetic rate (μmol m^{-2} s^{-1} CO_2)	Daily Net Carbon Gain (mol m^{-2} day^{-1} CO_2)	Carbon fixation during experimental monitoring period (mol m^{-2} CO_2/4 weeks)	Relative Change
Control group	2.47	0.213	5.964	-
0.5%Eu,0.1%Dy	2.60	0.225	6.30	+ 6%
0.5%Eu,0.25%Dy	3.52	0.304	8.512	+ 42%
0.5%Eu,0.5%Dy	3.29	0.284	7.952	+ 35%

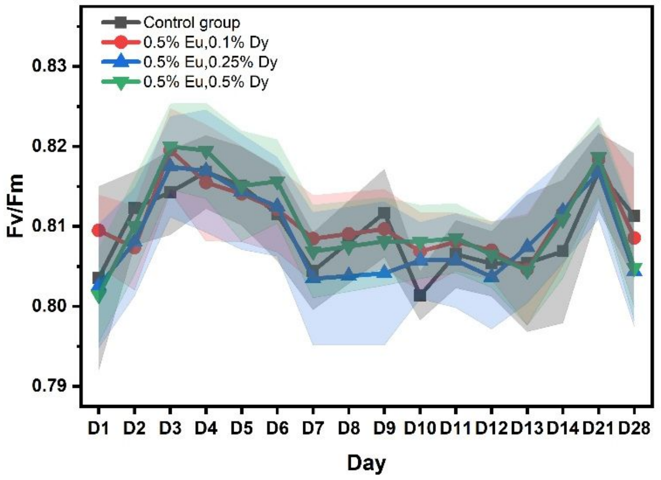

Figure S4: Maximum quantum yield of PSII (Fv/Fm) parameter of chlorophyll over 28 days for control and phosphor coated groups (The color-filled area is 95% CI).

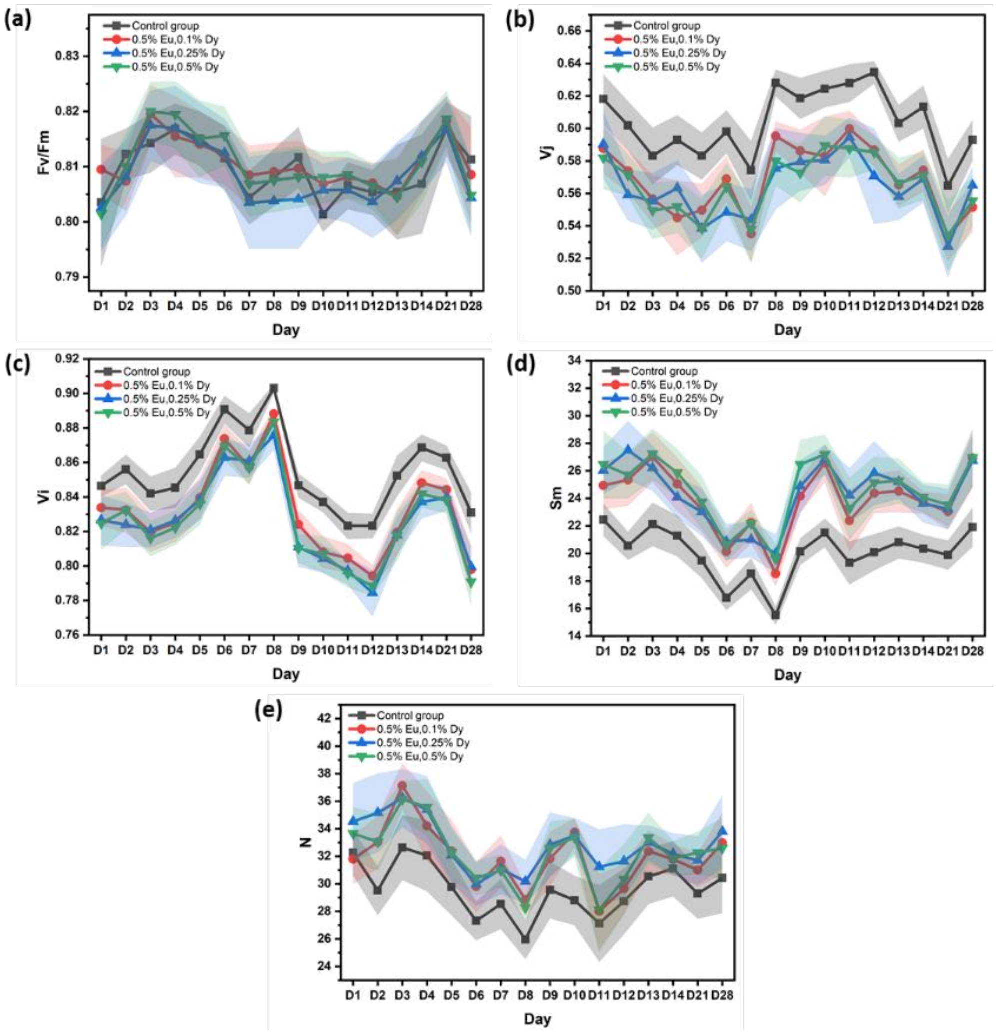

Figure S5: Plant health performance indicators from the OJIP curve (a) Fv/Fm is maximum

quantum yield of PSII, (b) Vj is relative variable fluorescence at J-step, (c) Vi is relative variable fluorescence at the I-step, (d) Sm is normalized area above OJIP curve, (e) N is turnover number of quinone acceptor reduction until maximum fluorescence, measured for 28 days.

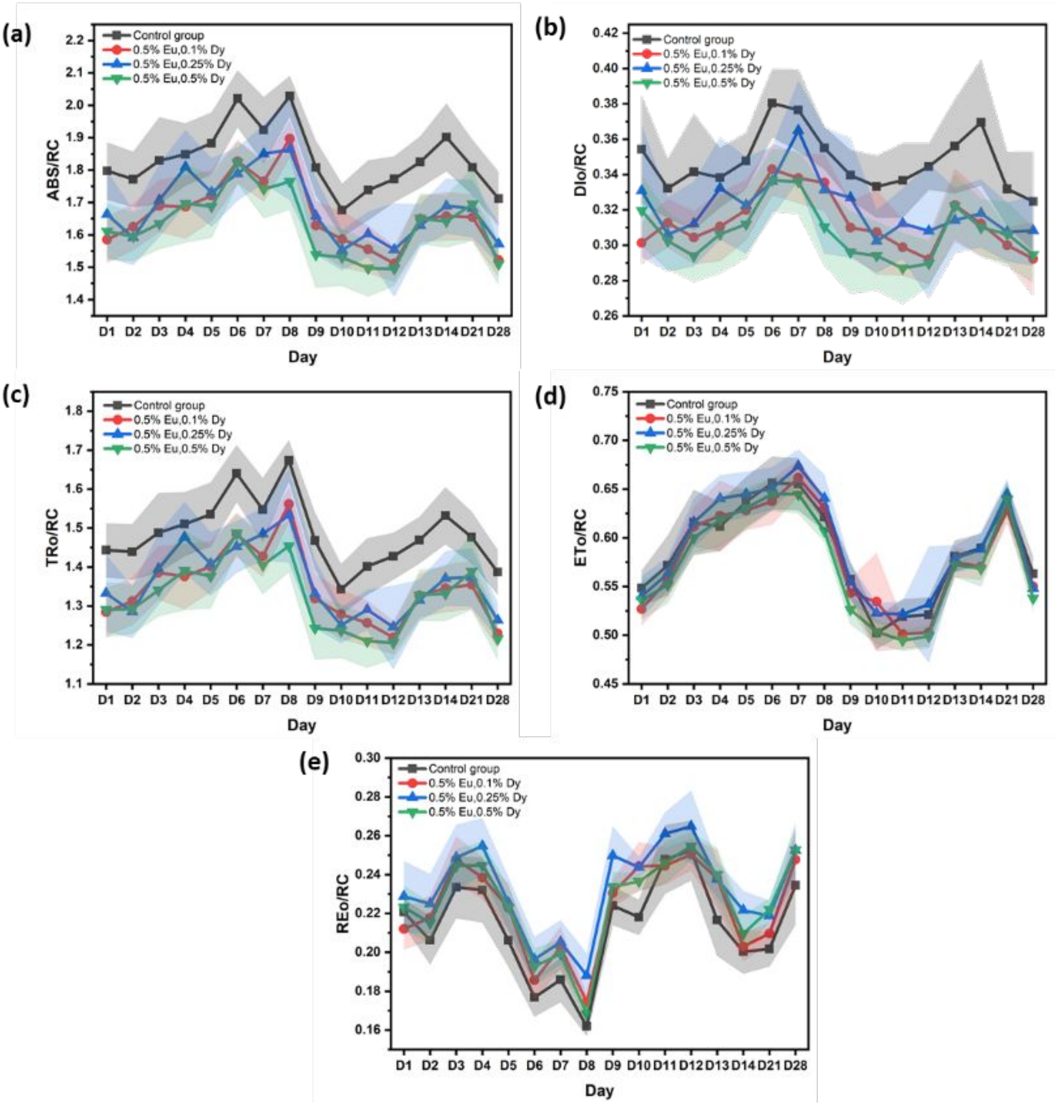

Figure S6: Plant health performance indicators (a) ABS/RC is absorption per reaction center, (b) Dio/RC is dissipation per reaction center, (c) TRo/RC is trapping per reaction center, (d) Eto/RC is electron transport per reaction center, (e) REo/RC is reduction of end electron acceptors per reaction center measured for 28 days.

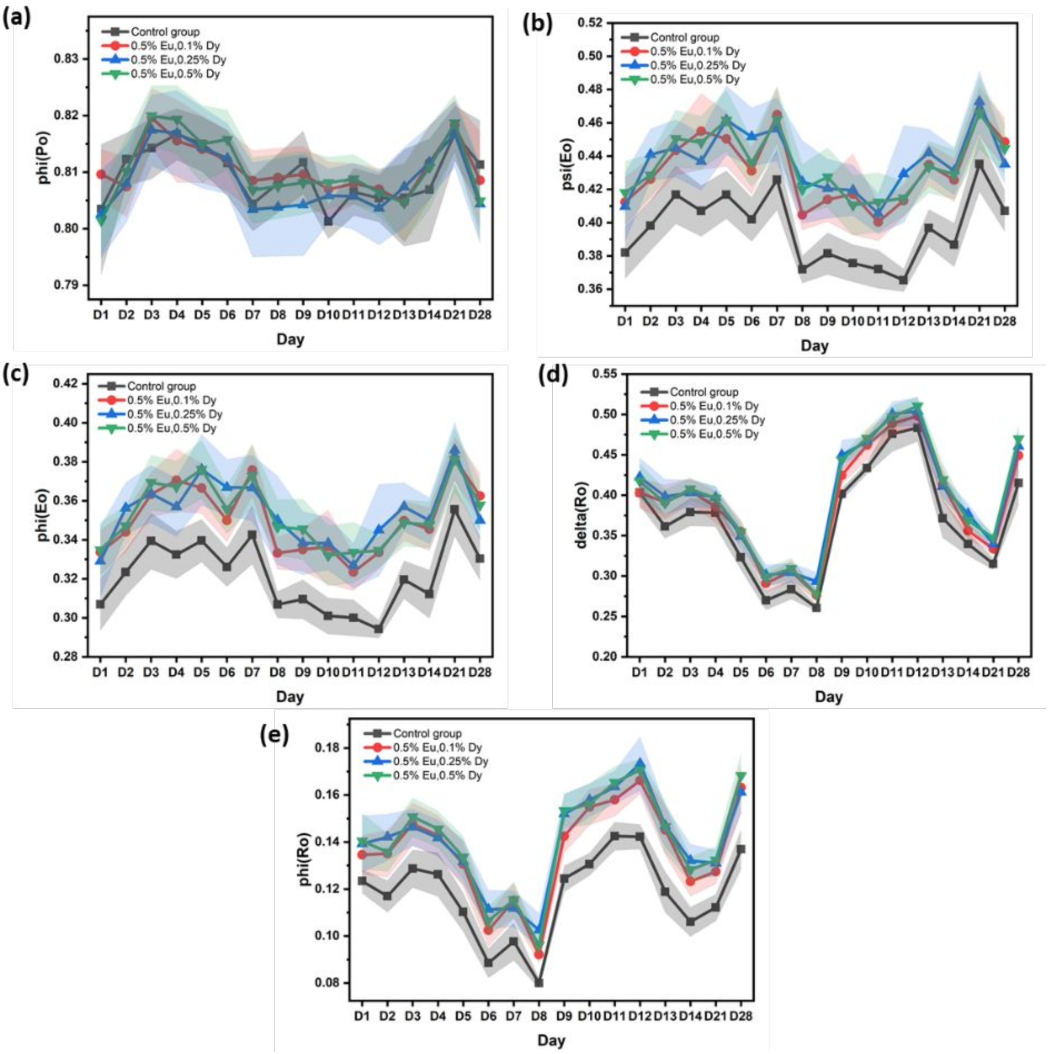

Figure S7: Plant stress, electron transport efficiency, photosynthetic efficiency under stress performance indicators (a) phi(Po) is maximum quantum yield of Fv/Fm, (b) psi(Eo) is electron transport efficiency, (c) phi(Eo) is photosynthetic efficiency under stress, (d) delta(Ro) indicates stress at PSI acceptor, (e) phi(Ro) integrates both PSII and PSI performance measured for 28 days.

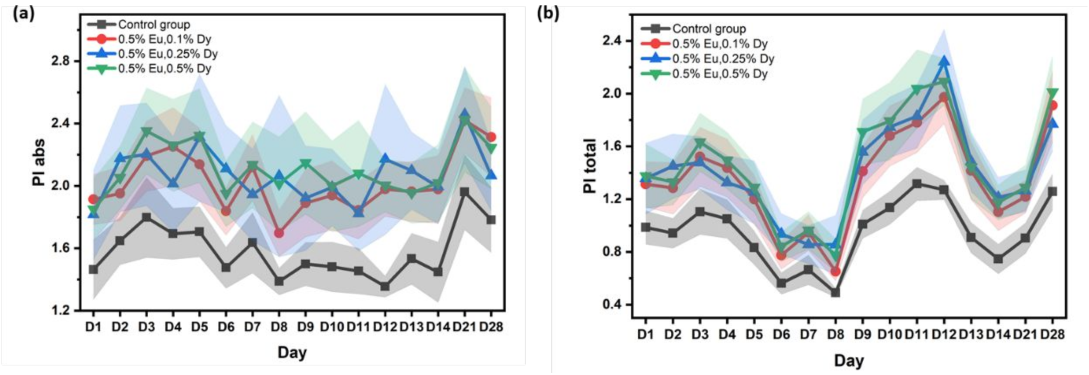

Figure S8: PI_{abs} and PI_{total} indicators measured for 28 days.
